# Endoscopic Staged Management of Large Pancreatic Walled-Off Necrosis in a Pediatric Patient

**DOI:** 10.7759/cureus.100405

**Published:** 2025-12-30

**Authors:** Mohamad Sabsabee, Abdullah Sabsabee, Khalid Bamakhrama, Buthaina Almurbati

**Affiliations:** 1 Pediatrics, Al Jalila Children's Speciality Hospital, Dubai, ARE; 2 Pediatrics, University of Kalamoon, Kalamoon, SYR; 3 Gastroenterology, Rashid Hospital, Dubai, ARE; 4 Pediatric Gastroenterology, Al Jalila Children's Speciality Hospital, Dubai, ARE

**Keywords:** git endoscopy, pancreatic necrosectomy, pancreatic psuedocyst, pancreatitis, walled-off pancreatic necrosis

## Abstract

Walled-off pancreatic necrosis (WON) is an encapsulated collection that develops as a late sequela of acute necrotizing pancreatitis, typically after four to six weeks. In children, large WON is rare but can be life-threatening due to secondary infection, multiorgan involvement, or compression of adjacent structures. Traditional surgical necrosectomy carries significant morbidity; however, minimally invasive endoscopic ultrasound (EUS)-guided interventions have emerged as a safe and effective alternative.

We report a 12-year-old boy with a history of obesity and fatty liver disease who developed severe acute pancreatitis complicated by ascites and pleural effusion. After initial recovery, he presented with persistent abdominal pain and was found to have a large multiloculated pancreatic collection measuring approximately 14 × 14 × 15 cm consistent with walled-off necrosis. EUS-guided cystogastrostomy was performed using a 15 mm lumen-apposing metal stent (LAMS), draining purulent material that cultured *Staphylococcus aureus* and *Streptococcus mitis/oralis*. Staged endoscopic therapy included repeated endoscopic necrosectomies, intravenous antibiotics, and total parenteral nutrition. The patient achieved near-complete resolution of the necrotic cavity following two sessions of necrosectomy and subsequent stent removal at six weeks, without surgical intervention. This case highlights the role of the endoscopic staged approach in managing large pediatric WON, demonstrating effective drainage and necrosectomy with low morbidity and excellent clinical recovery.

## Introduction

Acute pancreatitis in children is a recognized condition, though necrotizing pancreatitis and its sequelae remain uncommon, accounting for approximately 5-10% of pediatric cases [1,2]. Walled-off pancreatic necrosis (WON) represents a mature, encapsulated collection of pancreatic and peripancreatic necrotic tissue that forms typically four weeks or more after the onset of necrotizing pancreatitis; which when infected becomes a pancreatic abscess [3]. The pathophysiology involves enzymatic digestion, tissue necrosis, and inflammatory encapsulation, which distinguishes WON from simple pseudocysts [4].

In pediatric populations, the etiologies of acute pancreatitis include systemic diseases, medications, trauma, infection, and congenital anomalies [5]. Although rare, large WON - greater than 10 cm in size [6] - can lead to abdominal compartment syndrome, infection, or persistent systemic inflammation, contributing to high morbidity and prolonged hospitalization [7]. Historically, open surgical necrosectomy was the standard treatment; however, it carried significant postoperative complications, including pancreatic fistula, infection, and long-term exocrine and endocrine dysfunction [8].

Over the past decade, minimally invasive strategies, particularly endoscopic ultrasound (EUS)-guided cystogastrostomy and necrosectomy, have revolutionized the management of necrotizing pancreatitis. The European Society of Gastrointestinal Endoscopy (ESGE, 2018), North American Society for Pediatric Gastroenterology, Hepatology and Nutrition (NASPGHAN, 2022), and American Gastroenterological Association (AGA, 2023) all endorse a staged approach, starting with endoscopic or percutaneous drainage, followed by minimally invasive necrosectomy if required [9-11]. This approach minimizes tissue trauma, preserves pancreatic function, and reduces mortality compared to open necrosectomy.

This report describes the successful endoscopic staged management of a large WON in a pediatric patient, emphasizing the applicability of guideline-based endoscopic therapy to pediatric patients [8].

## Case presentation

A 12-year-old boy with a history of obesity and fatty liver disease presented with severe acute pancreatitis complicated by ascites and pleural effusion. He required pediatric intensive care admission with non-invasive ventilation for respiratory distress. No gallstones, structural abnormalities, or drug exposures were identified as etiologies. The patient was treated conservatively with intravenous fluids, antibiotics, and analgesia. After stabilization, he was discharged on pancreatic enzyme supplement and proton pump inhibitor therapy.

**Figure 1:**
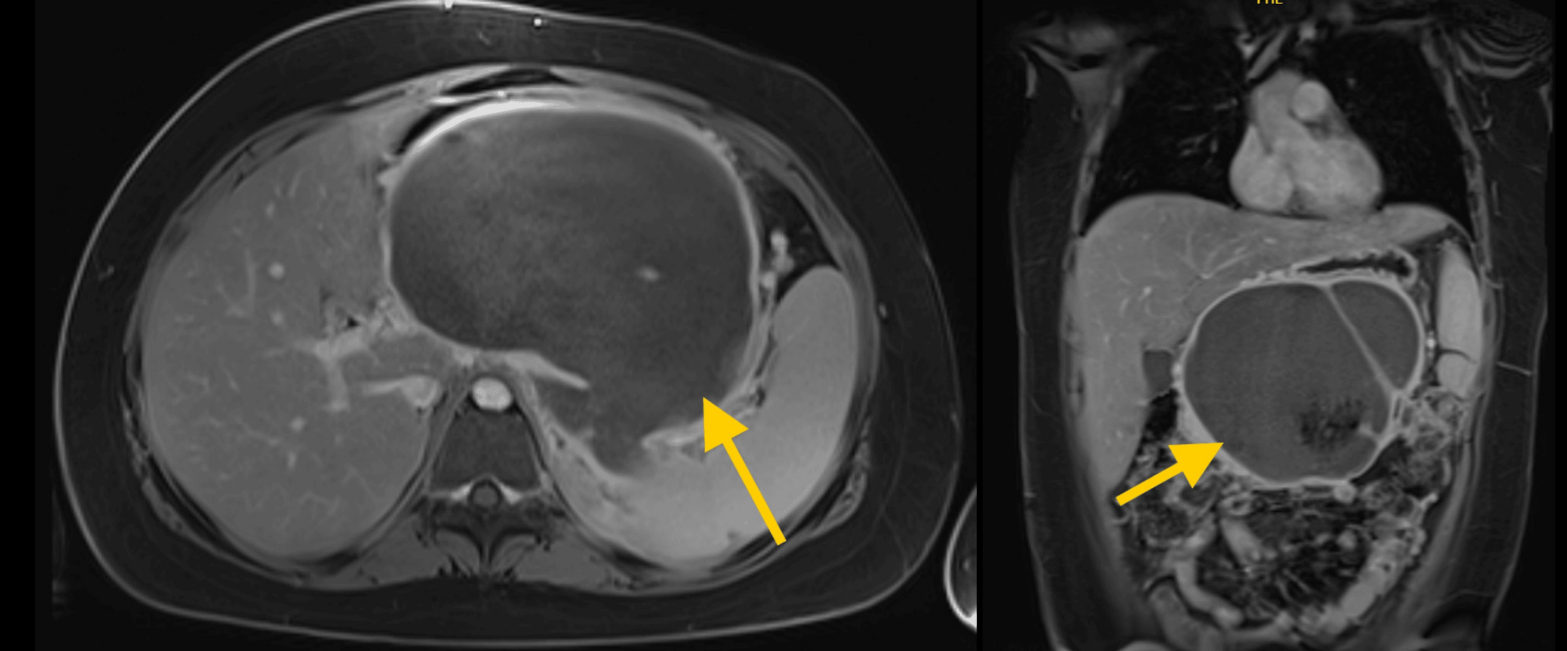
T1-weighted contrast enhanced MRI abdomen images axial view (left), coronal view (right) showing a large WON collection. WON: walled-off necrosis

Over the following months, the patient experienced intermittent abdominal pain and early satiety. Imaging revealed a large encapsulated pancreatic collection measuring approximately 900 mL, raising concern for WON. He was vitally stable, and laboratory tests revealed elevated pancreatic enzymes, as shown in Table 1. Magnetic resonance imaging demonstrated a multiloculated cystic lesion measuring 13.7 × 13.6 × 15.5 cm with internal septations and debris, consistent with a large WON as seen in Figure 1.

**Table 1:** Laboratory results CRP: C-reactive protein; PCT: procalcitonin; Hb: hemoglobin; WBC: white blood cells.

Time / Test	CRP (mg/L)	PCT (ng/mL)	Amylase (U/L)	Lipase (U/L)	Hb (g/dL)	WBC (×10³/µL)	Platelets (×10³/µL)
On admission (D1)	13	-	199	387	10	6.5	308
Post first necrosectomy (D7)	8.2	0.03	26	32	10.9	7.6	275
Fever onset (D11)	179	0.14	23	35	8.9	10.3	239
D19 of admission	128	0.11	-	-	9.2	9.5	304
Post third necrosectomy	15.1	0.11	-	-	10.9	7.8	462
Post stent removal	7.7	0.05	-	-	10.7	8.8	264

**Figure 2:**
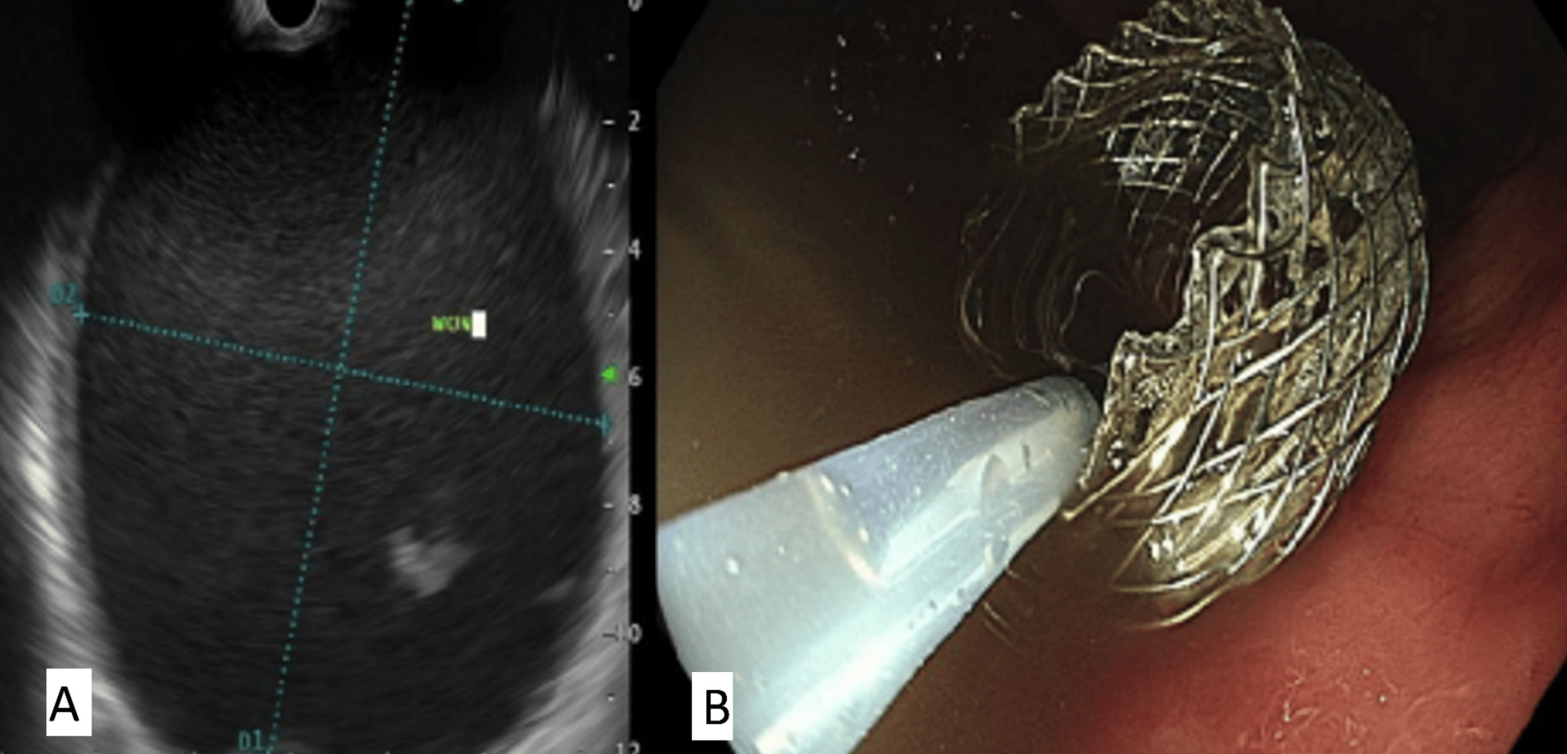
(A) EUS view of the WON on the right. (B) Endoscopic view of LAMS on the left. EUS: endoscopic ultrasound; WON: walled-off necrosis; LAMS: lumen-apposing metal stent.

The patient underwent EUS-guided cystogastrostomy with placement of a 15 mm LAMS (Hot AXIOS, Boston Scientific Corp., Marlborough, MA) as seen in Figure 2. Thick purulent material was drained, and culture grew methicillin-resistant *Staphylococcus aureus* (MRSA) and *Streptococcus mitis/oralis*. He was managed with proton pump inhibitor therapy, broad-spectrum antibiotic coverage with piperacillin-tazobactam, and total parenteral nutrition due to abdominal pain and vomiting.

**Figure 3:**
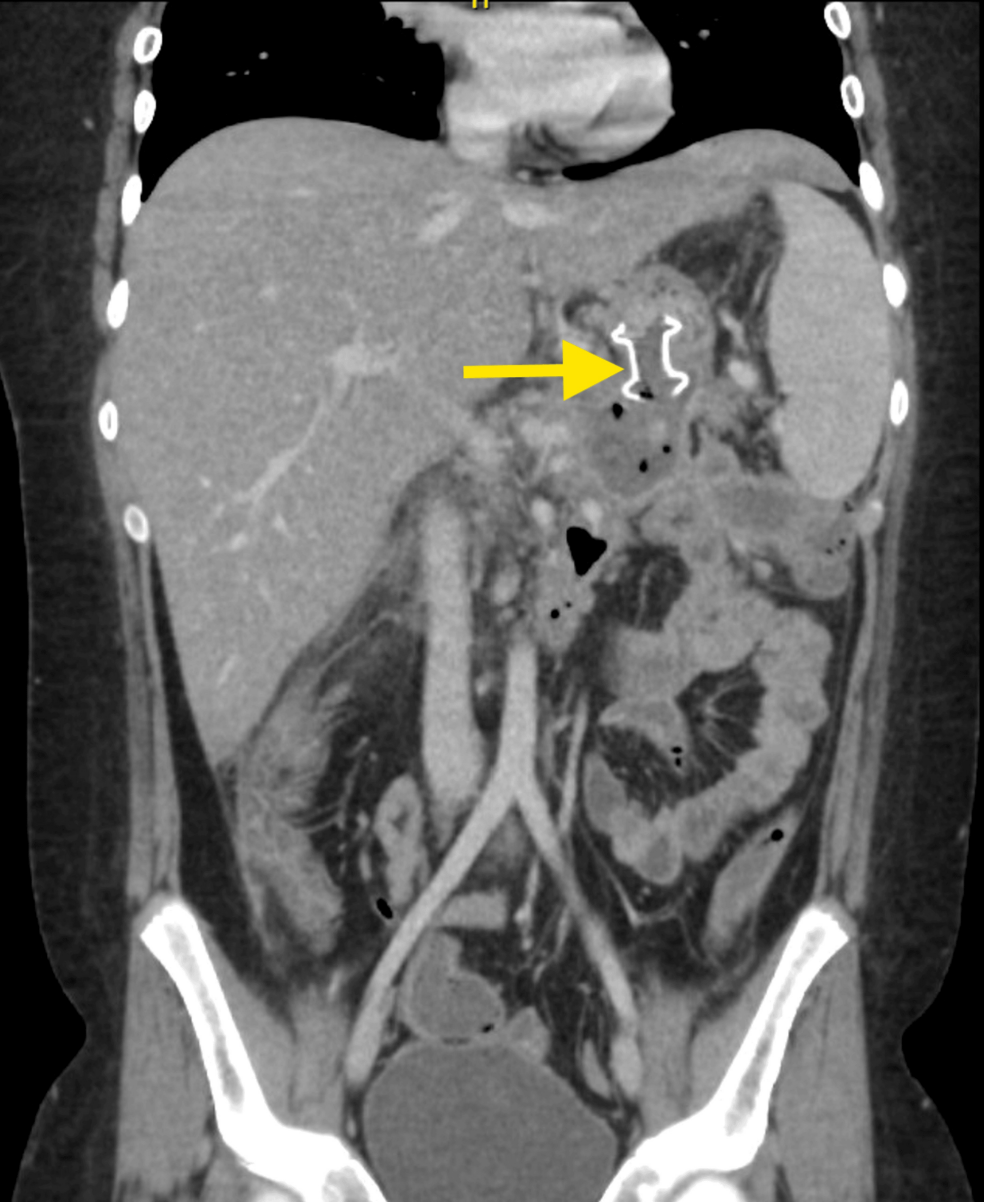
Axial contrast-enhanced CT abdomen image showing obstructed LAMS LAMS: lumen-apposing metal stent

A week later, planned endoscopic necrosectomy was performed under general anesthesia. The LAMS was patent, and extensive necrotic debris was removed using a snare and irrigation. Oral feeding was reintroduced gradually. However, the patient subsequently developed recurrent fever (maximum of 39°C) and abdominal pain. CT imaging revealed partial obstruction of the stent by debris and a persistent retroperitoneal collection, as seen in Figure 3. Laboratory tests revealed elevated inflammatory markers, as seen in Table 1. Antibiotics were escalated to vancomycin and meropenem.

**Figure 4:**
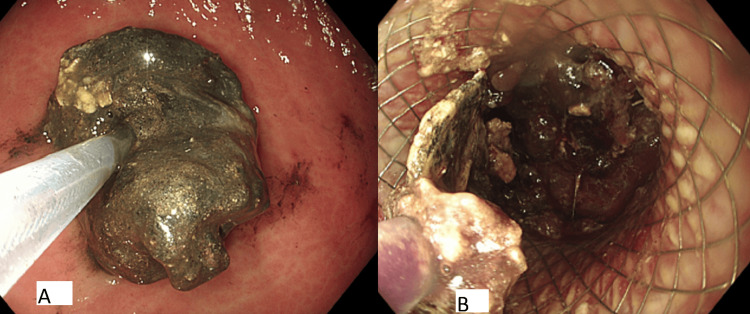
(A) Endoscopic view of obstructed LAMS. (B) Patent LAMS following necrosectomy. LAMS: lumen-apposing metal stent

A repeat endoscopic necrosectomy was performed two weeks following the first necrosectomy procedure. The cavity was accessed through the LAMS, and large fragments of necrotic tissue were extracted and suctioned as seen in Figure 4. Irrigation with sterile saline was performed until the cavity appeared clean. Minimal bleeding was encountered and controlled endoscopically.

A final endoscopic evaluation was performed two weeks following the second necrosectomy procedure. EUS demonstrated a nearly collapsed cavity with no discharge, and upper endoscopy confirmed a clean tract. The LAMS was removed without complication. The patient was discharged in stable condition on oral prophylactic antibiotics and pancreatic enzyme supplementation. A follow-up MRI of the abdomen was requested to confirm complete resolution and exclude recurrence, but it has yet to be done as the patient is lost to follow-up.

## Discussion

Large walled-off pancreatic necrosis in pediatric patients is uncommon but clinically significant due to diagnostic and therapeutic challenges. Unlike adults, where gallstones and alcohol predominate as causes, pediatric pancreatitis often arises from trauma, medications, viral infections, or genetic mutations [5,12]. In this context, delayed complications such as WON can lead to significant morbidity if not appropriately managed.

Current evidence supports the staged endoscopic approach as the preferred management strategy for symptomatic or infected WON. EUS-guided drainage provides direct transluminal access to the collection, enabling internal drainage and subsequent necrosectomy through the lumen-apposing metal stent (LAMS). Compared with open or minimally invasive surgical necrosectomy, endoscopic management offers lower morbidity, reduced hospital stay, and better preservation of pancreatic function [9,10,13].

In children, limited but growing evidence supports the safety and efficacy of this approach. A multicenter NASPGHAN study demonstrated a clinical success rate exceeding 90% and an adverse event rate below 10% for EUS-guided drainage of pancreatic fluid collections using LAMS [10]. Another pediatric case series reported favorable long-term outcomes without recurrence or significant complications [14]. The technical principles mirror those of adult protocols but require adaptation for pediatric anatomy, including a smaller lumen size and careful anesthesia coordination.

In this case, sequential necrosectomies were required due to stent obstruction, a common occurrence as necrotic debris sloughs into the cavity. Stepwise endoscopic clearance restored drainage and allowed for near-complete resolution without surgery. Nonetheless, follow-up imaging is important to exclude recurrence. Timely stent removal is essential, as most guidelines recommend removal after four to six weeks once imaging confirms cavity collapse to minimize the risks of delayed bleeding, buried stent syndrome, or gastric fistula formation [9,11,15].

Nutritional and antibiotic management are integral to successful outcomes. The patient was maintained on total parenteral nutrition during acute infection and transitioned to enteral feeds after sepsis control, consistent with current pediatric pancreatitis recommendations [16].

This case reinforces that EUS-guided drainage and necrosectomy can be safely applied in the pediatric setting when performed in specialized centers with appropriate expertise. Early multidisciplinary collaboration among pediatric gastroenterology, radiology, and critical care teams is essential for successful outcomes.

## Conclusions

Staged endoscopic therapy represents a transformative advance in the management of large pediatric WON. Through EUS-guided cystogastrostomy, staged necrosectomy, and structured follow-up, complete clinical and radiologic resolution can be achieved with minimal morbidity. Nonetheless, as the follow-up imaging was not done, we cannot exclude recurrence. This case underscores the applicability of adult-derived staged principles in the pediatric population and supports their adoption as the preferred standard of care in specialized centers.
